# Assessing safe and personalised maternity and neonatal care through a pandemic: a case study of outcomes and experiences in two trusts in England using the ASPIRE COVID-19 framework

**DOI:** 10.1186/s12913-023-09669-0

**Published:** 2023-06-22

**Authors:** Sarah Neal, Lucy Stone, Gill Moncrieff, Zoë Matthews, Carol Kingdon, Anastasia Topalidou, Marie-Clare Balaam, Sarah Cordey, Nicola Crossland, Claire Feeley, Deborah Powney, Arni Sarian, Alan Fenton, Alexander E P Heazell, Ank de Jonge, Alexandra Severns, Gill Thomson, Soo Downe

**Affiliations:** 1 Department of Social Statistics and Demography, University of Southampton, Southampton, UK; 2 Faculty of Medicine, Health & Life Sciences, Swansea University Medical School, Swansea University, Swansea, UK; 3 ReaCH Group, School of Community Health and Midwifery, Faculty of Health and Care, University of Central Lancashire, Preston, UK; 4 MAINN Group, School of Community Health and Midwifery, Faculty of Health and Care, University of Central Lancashire, Preston, UK; 5 Nursing, Midwifery & Palliative Care, Methodologies Division, King’s College London, London, UK; 6 School of Justice, University of Central Lancashire, Preston, PR1 UK; 7 School of Medicine, University of Central Lancashire, Preston, UK; 8 Newcastle Neonatal Service, Newcastle Upon Tyne Hospitals NHS Foundation Trust, Newcastle Upon Tyne, UK; 9 Maternal and Fetal Health Research Centre, Faculty of Biology, Medicine and Health, University of Manchester, Manchester, UK; 10 Department of Midwifery Science, AVAG/Amsterdam Public Health, Amsterdam University Medical Centre, Vrije Universiteit Amsterdam, Amsterdam, Netherlands; 11 Lancashire And South Cumbria Cardiac Network, Preston, UK

**Keywords:** COVID-19, Maternal health services, Midwifery, Crises, Case study, Organisational evaluation framework, ASPIRE COVID-19

## Abstract

**Background:**

The COVID-19 pandemic has resulted in profound and far-reaching impacts on maternal and newborn care and outcomes. As part of the ASPIRE COVID-19 project, we describe processes and outcome measures relating to safe and personalised maternity care in England which we map against a pre-developed ASPIRE framework to establish the potential impact of the COVID-19 pandemic for two UK trusts.

**Methods:**

We undertook a mixed-methods system-wide case study using quantitative routinely collected data and qualitative data from two Trusts and their service users from 2019 to 2021 (start and completion dates varied by available data). We mapped findings to our prior ASPIRE conceptual framework that explains pathways for the impact of COVID-19 on safe and personalised care.

**Results:**

The ASPIRE framework enabled us to develop a comprehensive, systems-level understanding of the impact of the pandemic on service delivery, user experience and staff wellbeing, and place it within the context of pre-existing challenges.

Maternity services experienced some impacts on core service coverage, though not on Trust level clinical health outcomes (with the possible exception of readmissions in one Trust). Both users and staff found some pandemic-driven changes challenging such as remote or reduced antenatal and community postnatal contacts, and restrictions on companionship. Other key changes included an increased need for mental health support, changes in the availability and uptake of home birth services and changes in induction procedures. Many emergency adaptations persisted at the end of data collection. Differences between the trusts indicate complex change pathways. Staff reported some removal of bureaucracy, which allowed greater flexibility.

During the first wave of COVID-19 staffing numbers increased, resolving some pre-pandemic shortages: however, by October 2021 they declined markedly. Trying to maintain the quality and availability of services had marked negative consequences for personnel. Timely routine clinical and staffing data were not always available and personalised care and user and staff experiences were poorly captured.

**Conclusions:**

The COVID-19 crisis magnified pre-pandemic problems and in particular, poor staffing levels. Maintaining services took a significant toll on staff wellbeing. There is some evidence that these pressures are continuing. There was marked variation in Trust responses. Lack of accessible and timely data at Trust and national levels hampered rapid insights. The ASPIRE COVID-19 framework could be useful for modelling the impact of future crises on routine care.

**Supplementary Information:**

The online version contains supplementary material available at 10.1186/s12913-023-09669-0.

## Introduction

The impact of COVID-19 on maternal health care has been both profound and complex. As well as the direct impact of infections on maternal and newborn outcomes [1] evidence is still emerging about the changes in access to care resulting in indirect impacts [2], and the various social and economic consequences [3]. Throughout much of the world, including the UK, the pandemic led to changes in access and quality of care because of specific policy decisions, pressure on services, and alteration in user behaviour due to concerns about infection risk.

There is increasing evidence on how COVID-19 has affected the delivery of reproductive care globally. Many services deemed “non-essential” were postponed or curtailed to reduce transmission and the strain on struggling health systems e.g. access to contraception and abortion were disrupted, particularly in low and middle-income countries [4]. A UK survey found that the majority of responding maternity organisations reduced the number of antenatal care (ANC) and postnatal (PN) contacts, using remote alternatives to face-to-face appointments [5]. Widespread restrictions were placed on companionship during ANC appointments, birth and the PN period [6].These changes resulted in poor care and distress for women [7]. Kotlar et al. [4] raised concerns that the pandemic resulted in changes to maternity services without evidence of benefit, and that some of these changes negatively impacted the health and rights of women, including reduced emotional support during pregnancy and labour, and reduced post-partum hospital stays.

However, there was marked heterogeneity in how these changes were introduced, as well as in the impacts both between and within countries. To date, there have been no studies of impacts at a system-wide level. The ASPIRE COVID-19 project, initiated in July 2020, was designed to examine what makes maternity and neonatal care safe and personalised in a pandemic, and beyond. The focus on “safe and personalised” reflects UK Government policy which is articulated in the “Better Births” National Maternity Review Report’s call for maternity care to focus on personalised care that priorises safety, continuity and the empowerment of women [8]. paper aims to develop a coherent and comprehensive understanding at the English National Health Service (NHS) provider level regarding the processes and outcomes of changes in safe and personalised maternity care before and during the COVID-19 pandemic. We present two case studies from separate health trusts (an NHS trust is an organisational unit within the National Health Services of England and Wales, generally serving either a geographical area or a specialised function and set up to provide goods and services for the purposes of the health service) and compare changes to resources, service delivery and outcomes that occurred before and during the pandemic to date. We applied a conceptual framework to the case studies designed to understand how COVID-19 (or indeed any other potential health system shock both within the UK and globally) impacts safe and personalised maternity care.

## Methodology

### Use of a case study methodology

Crowe et al. (2011) state that a case study methodology is “a research approach used to generate an in-depth, multi-faceted understanding of a complex issue in its real-life context,” which fits within the aim of our research. The method offers the opportunity to explore a phenomenon, policy, or situation from a range of perspectives. These characteristics make them popular for health systems research, which requires the analysis of multiple factors from the viewpoints of different stakeholders which can be placed over time within a changing context [9, 10], which again fits with our objectives. A case study has five key stages: defining the case, identifying the case, collecting and analysing the data, interpreting data and reporting the findings [9].

We used a mixed-methods organisational case study approach drawing on qualitative and quantitative data to triangulate our findings and provide greater depth and scope; total reliance on qualitative material would mean there was no possibility to include objective measures of change in service delivery and outcomes, whereas quantitative data alone would lead to areas being missed where no indicators were available, and an inability to explore the drivers and pathways of change and impacts on stakeholders. Our approach is essentially descriptive, with the purpose to describe a phenomenon in its real-world context. Adequate integration is key to an effective mixed-methods case study, and we adopted a convergent design where the qualitative and quantitative data were extracted and analysed during a similar timeframe (although the qualitative data had been collected earlier in 2021). Based on Stake’s definitions of case study purpose we suggest that our studies are defined as instrumental in that we use them to gain a broader appreciation of the impact of COVID-19 on the delivery of maternity care by trusts in England, but there is also a collective element as we compare data from both trusts to provide wider insights [9].

### Applying a conceptual framework for data analysis

**Fig. 1:**
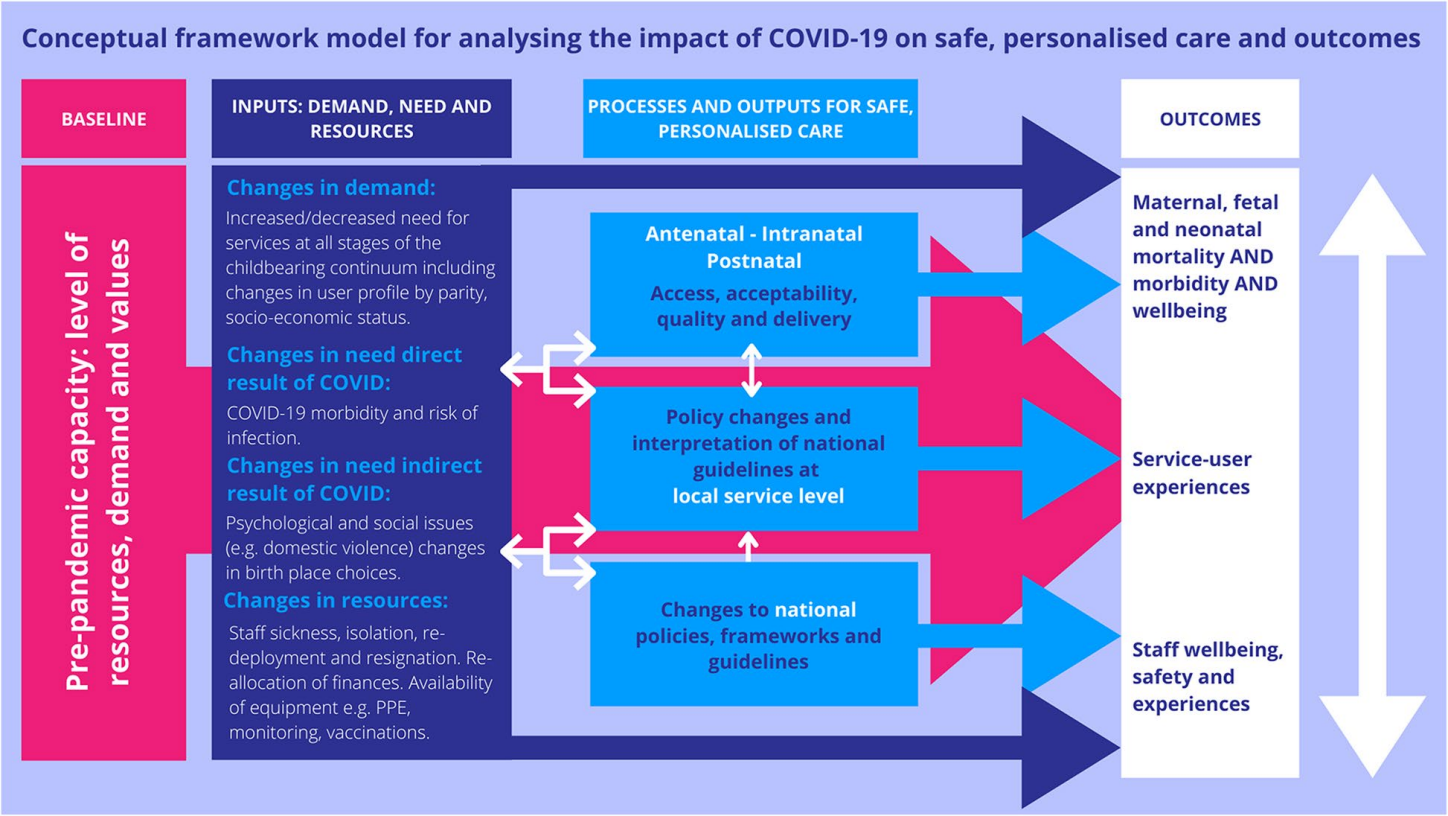
Conceptual framework for analysing the impact of COVID-19 (and other crises) on safe and personalised care

The case study analysis was structured around the ASPIRE trust-level conceptual framework (see Fig. 1) which was one of the wider outputs from the ASPIRE COVID-19 project. This framework was developed as an iterative process. Initial work drew on the growing literature on the impact of COVID-19 on health services, and led a temporal framework outlining the maternal health processes and outcomes that could be influenced by the pandemic. This was augmented by earlier work within the ASPIRE COVID-19 project that included thematic analysis of interviews across the seven trusts, interviews with national policy makers, survey analysis and analysis of policy documents, which enabled a more detailed framework which established pathways of potential impact [6, 11–13].

The framework suggests a starting point of the pre-pandemic baseline, and then a flow from inputs through to processes and health and wellbeing outcomes for both staff and service users. Directional arrows suggest paths of influence, and the possible influence of policy changes on demand, processes and outcomes are included. It is designed to be adapted to other future crises, or for analysis and development of routine maternity and neonatal care. Use of a framework provides a clear structure for comparing the two trusts. Each component of the framework was expanded and illustrated with trust-level data from the two case studies.

As part of the wider ASPIRE COVID-19 there was an attempt to define the components of safe and personalised care. Our initial starting point drew from how NHS England defined personal and safe care in March 2020 [14] as:

- Implementing best practice care, such as that set out in the Saving Babies’ Lives Care Bundle
- Rolling out Continuity of Carer
- Working as a multi-disciplinary team across maternity and neonatal services
- Reviewing and learning from incidents
- Taking part in the Maternity and Neonatal Safety Improvement Programme
- Ensuring women are supported to make decisions about their care and that these are recorded in a Personalised Care and Support Plan
- Making Maternity Digital Care Records available to all women
- Working towards 75% of Black and Asian women receiving Continuity of Carer by 2024, along with women living in the most deprived areas.

A further literature review using the National Maternity Review Report [8] as a starting point fed into a round table discussion with members of the ASPIRE COVID-19 stakeholder group, who represent leaders and policy makers in maternal health care from a range of backgrounds. These discussions fed into the development of the framework, as well as the process for choosing variables.

### Case study trust selection: defining and identifying the cases

The two NHS organizations are part of seven NHS maternity providers within England included in the wider ASPIRE COVID-19 project. These seven trusts were identified through a sampling plan that ensured diversity, depth and breadth of the target population. The sampling plan drew on macro-level factors known to impact maternal health (e.g. deprivation, ethnicity), meso-level factors according to health service organisation (Care Quality Commission – CQC-rating, facilities), and micro level factors such as parity.

Trust A is located in the north of England. It manages around 4,500 births each year across three sites. It services a mixed socio-economic population, with some areas among the top 25 most deprived in the country, while others are much more socially advantaged. Less than 1% of the population are from ethnic minorities. In 2018 Trust A had a CQC rating of “requires improvement.” Trust B is in the Midlands and manages around 2,500 -3,000 births a year, so smaller than Trust A. It is an area of low deprivation with around 7% of the population from ethnic minorities. Conversely this trust had a 2018 CQC rating of “outstanding,” providing a strong contrast. Neither trusts had level three neonatal intensive care provision. Both organisations were chosen for the case study because they were able to provide at least some timely and high-quality clinical and service-related quantitative data (there were difficulties accessing quantitative data for several the other trusts). These two trusts represent geographic, and to some extent socio-economic diversity, but we acknowledge these are not representative of UK maternity providers as a whole.

### Collecting and analysing the data

The case studies draw on both quantitative and qualitative data from a range of sources. trusts were requested to provide data on key quantitative indicators from routinely collected data. As there is no agreed list of variables to measure safe and personalised care, a list was identified through an initial analysis of key documents, in particular the National Maternity Review report which introduced these concepts into NHS policy [8, 15] and was also informed by the stakeholder discussions described earlier. They were then reviewed with the research staff taking part in ASPIRE COVID-19 at trust level to establish which indicators were accessible and available (see Appendix 1). The data were requested from the beginning of 2018 to allow an understanding of pre-pandemic trends, but was not always available for the full period.

Other quantitative data were taken from:

- The Family and Friends test [16]: a short, anonymous survey designed to help service providers gather user’s views on the service.
- Safe Staffing data [17]: Trusts are required to publish information about the number of registered and non-registered nursing staff and midwives working on each ward, as well as the percentage of shifts meeting ‘safe staffing’ guidelines.
- UK Government data on COVID-19 incidence [18]: These were taken from the official UK government website for data and insights on coronavirus
- NHS data on COVID-19 hospital admissions [19]. This was based on data compiled monthly.

For the qualitative component, 30 in-depth interview transcripts (half from each trust) from service users and staff were randomly sampled from 96 in-depth interviews undertaken in the included trusts as part of the wider ASPIRE COVID-19 study between March 2021 and October 2021. These semi-structured interviews explored the views and experiences of service users and staff across all levels of the maternity and neonatal services in relation to the organisational response during the pandemic. Interviews were sampled for pragmatic reasons related to time and resources, with one in three transcripts chosen from the ungrouped data repository for each trust until the required number were reached. Sampling was not based specifically on achieving data saturation as we were not undertaking an inductive analysis, but looking for processes and experiences that could explain components in the ASPIRE model or direct the quantitative analysis, or that might contextualise the quantitative findings. However, 30 interviews were chosen as while the issue is deeply contested, this is frequently suggested as sufficient for supplying sufficient depth [20].

Full details of the methodology for the qualitative data collection can be found in Appendix 2. Of these transcripts, 17 were from service users and 13 were from staff, with a total of eight staff being at managerial level. Transcripts were analysed to gain insight into experiences and perspectives relating to the changes and impacts of the pandemic, for both staff and service users. Additionally, we gathered documentary evidence from Board Reports and trust communications outlining changes in policies and practice.

Quantitative variables and findings from the qualitative interviews and documents were mapped against the conceptual framework. Quantitative data were analysed over time using appropriate graphic methods (line and bar graphs), and notable trends were recorded. Qualitative and documentary data sources were used to triangulate trends and identify information gaps, as well as identify discord and concurrence.

### Interpreting and reporting the findings

Findings were shared within the team (including those involved in collecting the qualitative data), and with wider members of the stakeholder group and research staff within the two trusts to discuss possible interpretations. Each case study was written up separately in report format to enable the first draft of the manuscript to compare findings from the two trusts.

#### Findings

Our findings are structured under sub-headings that relate to components of the framework in Fig. 1.

### Inputs: demand and need for maternity services in case study trusts

A crisis can directly change the level of need by changing the number of service users, intensity or type of services required. In the case of COVID-19, this may be either through the need to care for COVID-positive women within the maternity health care system or through the need for protective measures to prevent the spread of infection.

#### Direct changes in need: COVID-19 incidence and admissions

**Fig. 2:**
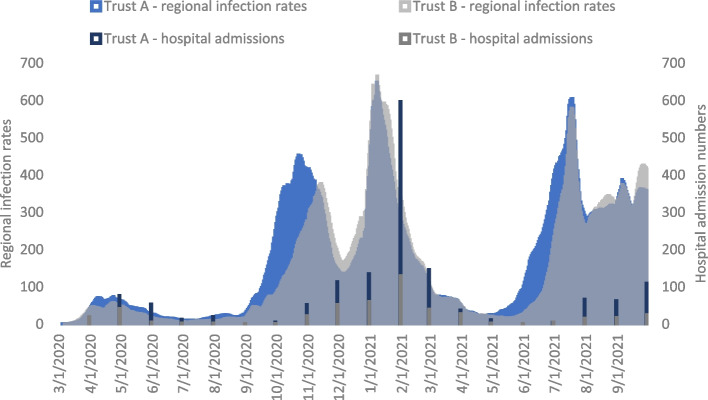
COVID-19 regional infection rates (7-day rolling rate per 100,000) and hospital admission numbers

An examination of monthly bookings and births, as well as the booking profiles by parity and decile, found there was no evidence of any change in demand for services occurring in either trust. We were unable to acquire information on the number of women testing positive for COVID-19 within the maternity health system, but analysis of regional incidence data and trust admission data enabled us to map the waves of the pandemic for both trusts (see Fig. 2). At the beginning of the pandemic, cases and admissions were fairly low in the locality of both case studies (although cases are subject to under-reporting because of a lack of testing services). After the first wave, there were then two spikes in cases, between August 2020 and February 2021 with a concurrent increase in COVID-19 admissions that peaked in January – February 2021. Following a dip in cases in March and April 2021, cases then increased again and remained high up to the end of September 2021, but in this wave hospital admissions did not rise so markedly. These data give some indication of when stresses are likely to be greatest on the health system, both in terms of cases, which will affect both the need for measures to reduce transmission and possible staff sickness and admissions that will increase demand on the hospital infrastructure.

#### Indirect need: mental health concerns

**Fig. 3:**
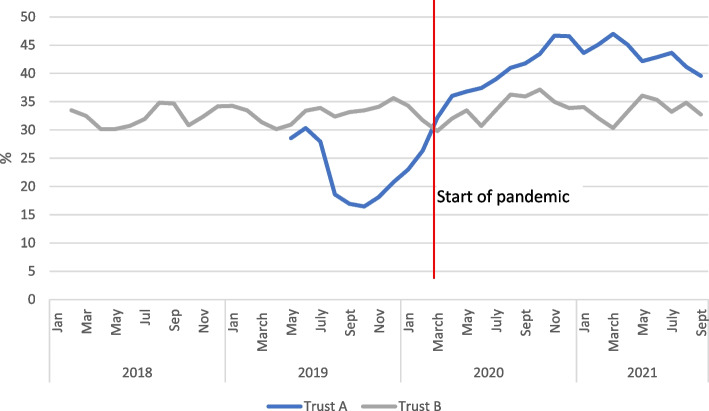
Percentage of bookings with mental health concerns, 2-month rolling average for Trusts A and B

**Fig. 4:**
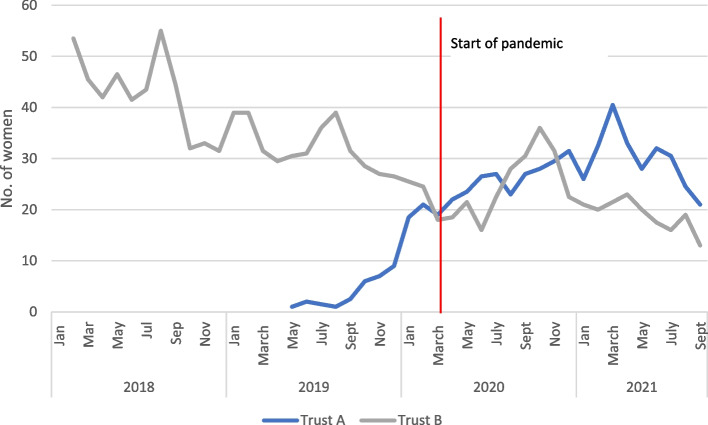
Number of women referred for mental health concerns after booking 2-month rolling average

A crisis can also produce indirect changes to the need for services. For instance, during the COVID-19 pandemic, there was a documented increase in intimate partner violence [21] and a risk of increased mental health concerns [22]. Both trusts acknowledged these increased risks in communications and guidelines provided to staff, but there were no data to track the incidence of domestic violence. Data were, however, available for both trusts related to mental health concerns at booking, and referral at any time during pregnancy to mental health services (see Figs. 3 and 4). Trust A shows a marked and consistent increase in reported mental health concerns (from a very low baseline) to a height of around 50% at the end of 2020. Referrals to mental health services at any time during the pandemic also increased from mid-2019. Trust B however, demonstrated no change in reported mental health concerns, and a decline in referrals.

As it is unlikely there will be such marked differences between regions in mental health and wellbeing, it is likely that to some extent patterns reflect the way midwives identify women with mental health concerns and the availability of specialist referral facilities. The increased identification and referral in Trust A could be due to strong perinatal mental health services in Trust A, which appears to have prioritised this area. Maternal anxiety was described in the qualitative data for both trusts. Trust B did acknowledge the issue since they introduced a system through which inpatients could indicate if they wished to talk about anxieties or concerns.

### Inputs: resources

#### Staffing

**Fig. 5:**
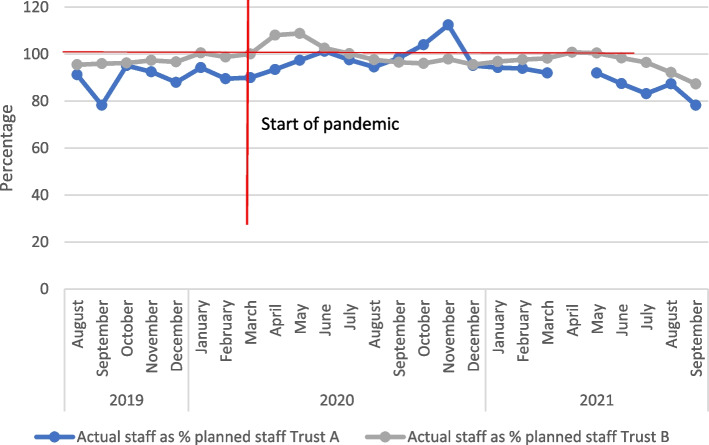
Data for registered midwives (combined for day and night staff and across sites) fill rate: actual staff as % of planned staff for Trusts A and B

The COVID-19 pandemic saw rises in staff sickness and self-isolation and redeployment in both trusts. Data from the safe staffing datasets (which provides information on actual staff hours worked versus those estimated as required) for both trusts showed that before the pandemic in Trust A there was already a staff shortfall for registered midwives, while for trust B this was much less marked. Both trusts saw an increase in staffing for registered midwives in the early phase of the pandemic (see Fig. 5), which may reflect the national drive to increase the NHS workforce at that point. In Trust A, the increased staffing levels continued until around the end of 2020 (although the increase was inconsistent), but then reverted to pre-pandemic levels before summer 2021, subsequently falling further.

In Trust B there was a shorter period of increased staffing (March-June 2020) before returning to pre-pandemic levels, and then figures showed a marked decrease from June 2021 onwards. Data for non-registered staff (e.g., support workers) was more inconsistent, particularly for Trust B (possibly due to small numbers not shown), but Trust A again showed a marked decrease in staffing from around June 2021. No data were available on trends in other health professionals.

Although the quantitative data suggest a more concerning situation in Trust A, the qualitative data from both trusts highlighted the negative impact of staff shortages. Several responses from Trust A acknowledged that staffing was a long-term problem pre-pandemic and not specific to the COVID-19 situation:

I think, to be quite honest, the pandemic’s come at a time when we were probably at a national shortage of midwives. We had stress before. Eighteen months ago, we were short of staff. So, you know, so even probably before the pandemic hit, I think we were short of staff. So, it just cemented it even more, really, I think the unit was run on goodwill, and I think to a degree, it still is. Health care provider, Trust A.

While Trust B’s staffing figures may not have looked too concerning until mid-2021, the qualitative evidence suggests that staffing levels were maintained at some individual cost to health care providers who felt morally obliged to work more hours than they wanted, with detrimental effects on their mental wellbeing:

“I can remember walking into work literally in tears thinking, what am I doing going in here? You know, I'm retired. I shouldn't be coming in. You know, I'm bank [temporary staff]. I shouldn't really be working here. But I also knew from a, from the point of view of staffing, having been the coordinator, how you reliant on your bank, everybody to turn up (…). So, I felt a moral obligation to come in and I do enjoy going to work but of recent I do feel very anxious because again, because of the staffing …” Health care provider, Trust B.

When asked what is needed to ensure safe and personalised maternity care in the future, most respondents said that the answer lies in safe staffing. Interview data indicate that women also noted the low staffing levels, particularly on the postnatal ward.

#### Access to resources

Beyond staffing levels, concern with excessive bureaucracy was reported as a way to free up time to care for front-line care providers and to create conditions for system agility e.g. making resources available without spending excessive time negotiating opaque organisational systems or being denied requested resources altogether. A reduction in bureaucracy early on in the pandemic was one of the few positive factors raised across several sites in the wider ASPIRE study, which meant they could rapidly access resources that had previously been tied up in ‘red tape’. Streamlined processes meant they could access equipment needed, quickly and easily:

“We bought, we used every resource we had. So, and actually, what was quite liberating at the beginning was everything was, you can have the resources there, you can have this, you can have it, whatever you need to preserve life, you can have (…). And actually we have the permission to do that, how we always want to run the service, you know, and that was quite liberating. And, you know, you weren’t questioned because it was like, if that's what you need.” Manager, Trust B.

### Processes and outputs for safe and personalised care

In terms of the framework, changes in demand/need and resourcesare likely to directly impact on the processes and outputs for safe and personalised care. However, changes in policy and guidelines either at national or provider level may also, in turn, bring about more structured changes in delivery of care such as new advice on criteria for interventions or restrictions on birthing options. Changes in processes and output are hypothesised to affect access, acceptability, quality and delivery of care across antenatal, intrapartum and PN care. Our case studies produce a wealth of information on a range of components of care in line with the framework. Here, we focus on summarising key findings that illustrate the pathways to change.

#### Processes of ANC

In the case of ANC, both Trusts implemented policies to carry out some contacts remotely. Neither seemed to set a particular target for the proportion that should be remote, although Trust B produced advice about which visits could be remote and Trust A published public-facing guidance saying appointments would be remote where possible. Available data suggest that the percentage of appointments delivered remotely was low in both trusts: the highest proportion of remote contacts in any month was 12% in Trust A and around 10% for Trust B.

Possibly more importantly than the way services were accessed, the percentage of women who received fewer than six ANC contacts (the minimum recommended by the Royal College of Midwives) increased in both trusts, with a revised and reduced schedule of ANC introduced in Trust B (although still with a recommended minimum of over six visits).

**Fig. 6:**
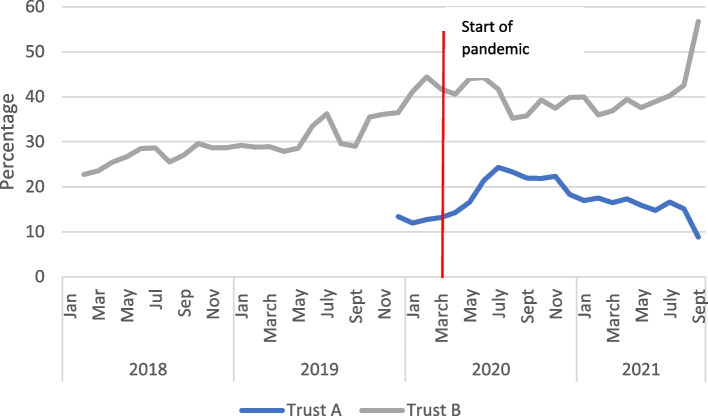
Percentage of women having less than 6 ANC contacts as % of all births, 2-month rolling average

As the pandemic progressed, in Trust A the patterns show an increase in the percentage of women experiencing less than six ANC contacts in the months after the start of the first wave (see Fig. 6). This percentage then decreased, whereas in Trust B (which had a much higher percentage of women receiving less than six ANC contacts than Trust A) there is a steady increase in the percentage of women receiving less than 6 ANC contacts *before* the start of the pandemic, followed by a sharp increase in the percentage of women receiving a lower number of ANC contacts from September 2021. This suggests that the number of ANC contacts may have been reducing in Trust B before the pandemic. Changes in Trust B may be less related to changes made in service delivery as initial responses to the pandemic, but the ongoing crisis may have exacerbated a situation already in existence. There was no clear pattern of change for gestational age at first booking for either trust.

It should be highlighted that here the routinely collected quantitative data contrasts with the experiences and perspectives of service users and staff. The interview data suggest that many women experienced remote appointments, particularly at the beginning of the pandemic, and that some staff were significantly concerned about the impact of this on both the psychological and physical health of service users. Interviews with both staff and service users in the trusts indicated a feeling that this potentially reduced the quality of service and raised levels of anxiety, and, along with fewer ANC contacts, raised concerns about problems not being adequately detected.

All of it is over telephone conversation and it isn’t unheard of, even at 16-weeks they still haven’t seen their midwives and they’re coming through at twenty-four, twenty-eight weeks, having not seen an actual midwife, only had a virtual contact. When you speak to the patient for them, for some of them who've had babies before they go, oh, it’s very different, but for those new mums, first-time mums find it’s difficult. They are quite anxious, the not knowing and because obviously it's all new, it makes it very difficult for them. Health care provider, Trust B.

I had less face-to-face appointments with midwives. It was telephone appointments or just a couple of face-to-face. I wasn't able to go and look around the hospital to see the labour ward or anything like that, all I could do was look on the website. But I would say it was more like, I couldn't really go and hear the baby's heartbeat. I think it was that, it was that lack of face-to-face contact with the midwife team that I found the saddest. Service user, Trust B.

#### Home births

Choice of place of birth is integral to persoinalised care. In terms of home birth, the policy responses of the two trusts played out differently. In both trusts overall numbers of home births were low, but in Trust A the initial response was to suspend home births for several months. However, they were reinstated after pressure from service users:

I think if I remember correctly, we stopped the home birth. We stopped the home birth. And then there was a serious objection came from the patients. And then our senior management was questioning that decision because from a PPE [personal protective equipment], you know, thinking from a patient point of view, what they said is that home is safer than your hospital, so we want to deliver at home. So, if I remember correctly, I think we went back and said, it is OK, we will do it. But the initial reaction was to stop it. Manager, Trust A.

Trust B, however, actively promoted home births as safer in terms of infection control. As a result, the trust provided extra training and support to enable the increased and more seamless provision of their home birth service.

And we were saying, right, let’s suspend the home birth service …. suddenly our head of service said, hang on a minute, we need to turn this on its head. We need to, where's the safest place for people to be at home, so we need to keep them at home … … And so, then we decided actually let’s shift the priority. So, let’s… let’s … because what we actually did was expand our home birth service at that point. Manager, Trust A.

**Fig. 7:**
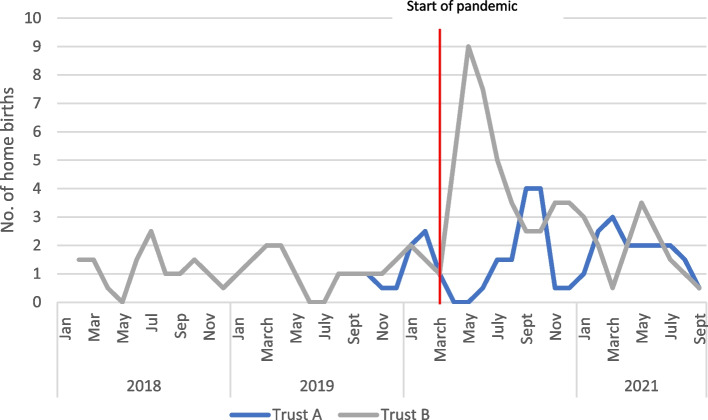
Actual number of home births in Trusts A and B, 2-month rolling average (actual numbers were used as were such a small proportion of overall births)

Quantitative trends are based on small numbers (monthly numbers per trust ranges from zero to nine across the period of study), but the data do demonstrate a cessation of home births for several months in Trust A followed by a modest increase, in contrast to a sharp increase from the outset of the pandemic in Trust B followed by a gradual decline (see Fig. 7). These differences occurred under the same national guidance, which recommended continuing all options for place of birth unless specific criteria of low staff or lack of availability of ambulance services were met [23].

#### Induction of labour

**Fig. 8:**
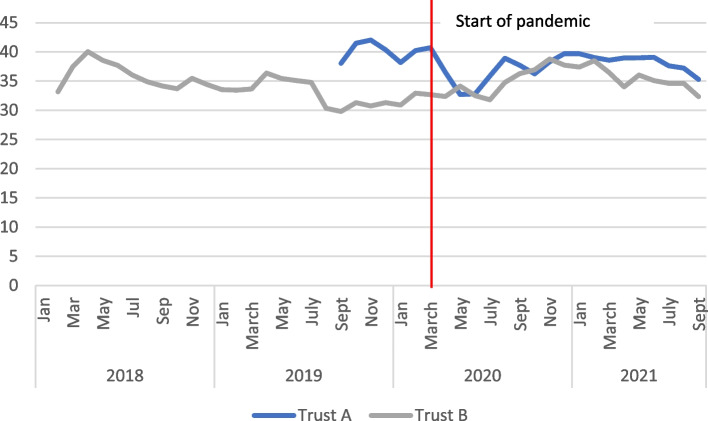
Percentage of births started using induction (including attempted induction), 2 month rolling average

Another example of diversity between the two trusts was the process of induction of labour, which clearly impacts both safety and personalization of care. Early in the pandemic the Royal College of Obstetricians and Gynaecologists issued guidance to avoid induction of labour “for indications that are not strictly necessary” [24]. Trust A showed a marked fall in inductions that may have been in response to this guidance, whereas Trust B shows a slight increase (see Fig. 8), which is supported by qualitative evidence from one interview suggesting some inclination to move births forward to protect vulnerable pregnant women from the increased risk they face from COVID-19.

Obviously, the third trimester was the point at which they were vulnerable. I wouldn’t say I was inducing earlier, but I would say and I don’t think I'm alone in this, but I think a lot of us had a mind-set that let's get them delivered and then they're no longer pregnant and their risk profile reduces, you know, because obviously the outcome, pregnant women on ICU were the worst. So, that was something I was very aware of, I wanted those women delivered. Health care provider, Trust B.

#### Postnatal care

**Fig. 9:**
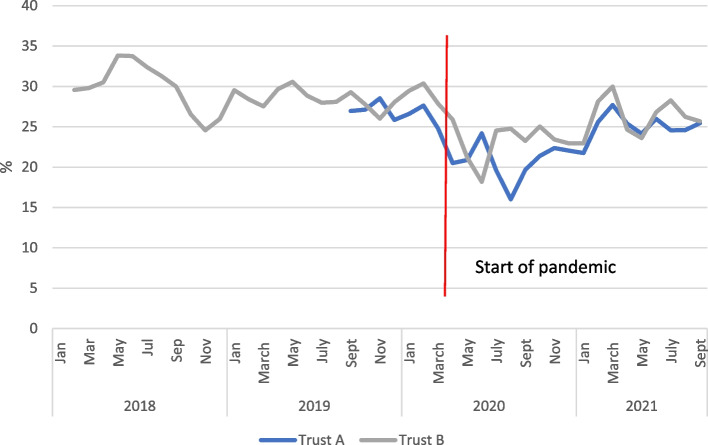
Percentage of women who had a length of PN stay more than 48 h, 2 months rolling average

Quantitative data for both trusts showed a reduction in the length of time women spent in hospital after the birth, with a decrease in stays over 48 h (see Fig. 9). This does not seem to be the result of a specific policy, but a combination of staff concerns over the risk of COVID-19 transmission and women discharging early, largely due to restrictions to partners being present. Staff raised this as a potential safety issue, and limited postnatal care clearly affected levels of personalization.

The staff obviously were very anxious about visitors on the ward. And, you know, in a bay of six once you have got another six partners and six babies your air exchange has completely gone down, and it was very difficult. We were often stuck between a rock and a hard place, you know, and the women didn’t want to stay in very long. So, we were worried about the impact of that you know, would the success of feeding be OK? You know, we kept thinking we were getting more wound infections, but we haven’t actually, you know, seen that evidence. it felt like we were getting a lot of people coming back in postnatally with problems but, you know, on audit, it doesn’t look like, you know, that was a correlation, although it felt like it. Manager, Trust B.

Women reported challenging experiences on the PN ward including reduced or restricted partner visits, a lack of support by staff and general confusion about what was going on in the ward.

If I could change anything, it would be once you’d have the baby. And I totally understand that with COVID, visitors can’t stay too long. But forget the visitors. I just think the care, I would make it so that I felt more looked after, more reassured, helped more with feeding. And just have that whole, it’s such a … nothing can prepare you for it. I thought “Oh, I know what to expect”. You just don’t and that there with this newborn life and you’re on your own, pandemic or not, you want people that are the professionals. And not make you feel like you’re an inconvenience and they haven’t got time for you, which is, I don’t think it’s any fault of their own. I think maybe they’re understaffed. But it wasn’t a positive experience afterwards, and it could have been so easily better. Service user, Trust B.

**Fig. 10:**
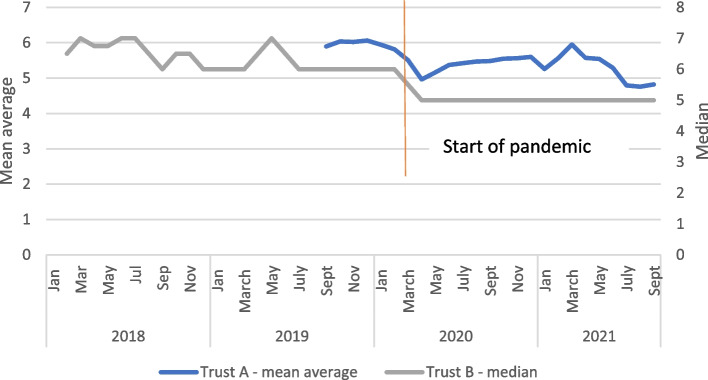
Number of PN contacts, mean for Trust A, median for Trust B, 2-month rolling average

The number of PN contacts reduced in both trusts from a mean average of around six before the pandemic in Trust A to an average of 5.4 during the pandemic, whereas the median number of visits in Trust B fell from between six and seven pre-pandemic to five during the pandemic (although again there was evidence of reducing numbers pre-pandemic: see Fig. 10). In Trust A they rose somewhat after an initial sharp drop at the start of the pandemic but fell again in early 2021. It is worth mentioning that there was some uncertainty in what was meant by a “PN contact”: these numbers are greater than might be normally expected from community post-discharge contacts and suggest that contacts on the ward before discharge were also included which means these data should be interpreted with caution. However, as the mode of data collection is unlikely to have changed at this time it is not unrealistic to think that these trends represent a change, even if it is not possible to separate hospital and home- based contact. Increased reliance on phone contacts caused concern for both staff and service users.

So, they normally get a day three visit for feeding, to keep breastfeeding, and that was done over the phone. And the discharge visit is usually done over the phone now, which I think probably has impacted on our breastfeeding rates and readmission rates and infection rates. Service provider, Trust A.

#### Readmission to hospital

Fears about safety were raised by staff along with concerns about readmission: while an audit did not suggest any increase in readmission in Trust B, an analysis of the incident reports for Trust A showed a potential increase. Neonatal readmissions increased from approximately five reported incidents per quarter just before the start of the pandemic to 13 incidents in quarter two of 2020, remaining at an average of nine throughout 2020 (see Appendix 3). This coincides with the first wave of the pandemic, as well as a fall in PN care during the pandemic, reaching a maximum of eight reported incidents, up from an average of three just before the pandemic. However, as there were also obvious sharp increases in readmissions in 2018 (particularly for babies), this situation cannot be attributed with any certainty to the pandemic per se.

#### Companionship and neonatal visiting

Companionship is a key area of personalised care. For many women and healthcare professionals, one of the most impactful changes to maternity services were the restrictions on partners and other visitors throughout the maternity care journey. This was as a result of national and local policies [25], but these tended to be interpreted somewhat variably, particularly during birth. No quantitative data were available on this, but qualitative data graphically illustrated how women had to attend antenatal (AN) appointments, including scans, alone, which reportedly left their partners anxious and distanced from the experience.

Things like the scans and as well having a partner just missing from, he wasn’t part of the birth, apart from showing up at the end, for a couple of hours. You know, he wasn’t part of it, of the whole thing. But yeah, I think it had a huge effect. So things like my 12-week scan, that’s one thing that sticks heavily in my mind. I previously had miscarriages, so I’ve had two miscarriages in the past. My last time I’ve had a 12-week scan, I found out I was going to miscarry. So when I went to the scan and he wasn't allowed in and I just sat in tears with these complete strangers who were lovely and did their best. But they … they knew that they weren't what was actually needed in support terms. My partner was sat in the car park waiting for me. Service user, Trust A.

For consultant appointments in particular, women felt that they would have benefited from having their partners' input in decision-making, and to help them take in and process information. Although women reported concerns about not having their partners with them for labour, they did have companionship during the birth itself (although it wasn’t clear at what stage of labour this became possible).

Views on the impact of PN restrictions on visitors showed a division between users and staff. Some service users felt vulnerable and isolated without their partner there to support them emotionally and physically with their new baby. However, some staff members felt that reduced visiting on the PN ward had been beneficial to women, in terms of breastfeeding and bonding, and for staff, because they had fewer additional matters to attend to.

One participant described the difficulties her family experienced when their baby had to be admitted to the Special Care Baby Unit (SCBU) immediately following birth, whereby partner restrictions compounded the trauma of being separated from her baby, not knowing what condition she was in.

They said, well, you’ve got to go home now, and I was like, hang on a second, I’m like, you know, I can’t move, so I couldn’t go be with her. She was all alone, just born, in SCBU, awful. You know, oxygen, everything all over the place and all tubes and everything. And then [name] had to leave immediately and he had to go home and we were just, I couldn’t move. She was in SCBU like who knows what, we didn’t know yet what had happened to her. So we didn’t know she had [condition]. We didn’t know if she was going to recover, if she would die, like who knows, like we didn’t know. Service user, Trust B.

### Changes in outcomes through the pandemic

In the framework, outcomes have been divided into maternal, fetal and neonatal mortality, morbidity and wellbeing, service user experience and staff wellbeing and safety. These can be influenced either through changes in the delivery of safe and personalised care, or more directly as a result of changes in demand and need: for instance, COVID-19 infection in pregnant women may lead to a high risk of poor outcomes even when quality of care is maintained.

At trust level, it is not realistic to examine trends in mortality for women and neonates as cases are rare, and these are not likely to be responsive quantitative indicators of changes in service quality unless conditions deteriorate to a catastrophic degree. There was no evidence of changes in gestational age at birth (although the data shows marked variability between months, making it difficult to ascertain trends). There was a possible slight reduction in babies with birth weights below 10% the last quarter of 2020, but this was quite unconvincing and could be a data anomaly. However, it is interesting to note that this trend has been found in national-level data in several countries including the UK [26, 27]. It is notable that, as mentioned earlier, there were increases in readmission rates, as well as increases in the previously undiagnosed incidence of small for gestational age (SGA) in Trust A, which could potentially reflect shortcomings in PN support and ANC which may warrant further investigation (see Appendix 4).

#### User experience

There is little quantitative evidence on either user or staff experience. The Family and Friends test was suspended for most of 2020, and when reinstated uptake was low. Qualitative evidence suggests service user experiences were mixed. Positive experiences included instances where health professionals had time for women and where there was constant provision of information. The difference that the individual midwife, doctor or health visitor can make was notable, and there is clearly appreciation for staff who went “the extra mile” in the face of acknowledged constraints. Several service users, however, suggested that their care was more cursory and less personalised than with previous births.

So, you know, there was a lot of communication. I didn’t feel that that was missing or that because of COVID, that they didn’t have enough time to talk these things through with me. I felt, I do, I say this all the time, the care that I got what I would have expected, even if COVID wasn’t on. So, with all those extra steps in place, worked really well. Service user, Trust A.

I definitely wasn’t seen as often as I was with [name], that’s for sure. Yeah, it was very short and brief on the on the times you know, that we did. Yeah, I did see them basically, I remember when I had [name] there was a lot more in-depth like questions and going through things and measurements and all that kind of stuff, whereas this was like, are you OK? Yes. OK, let’s hear where baby is, brilliant, your urine’s fine, crack on off you go. So it was, yeah, it was much more kind of like in and out. Service user, Trust B.

#### Staff wellbeing and safety

Data are not routinely collected about staff wellbeing and safety. Information from Board reports, while not reported separately for maternal health staff, reported high proportions of staff sickness caused by depression, anxiety, and other mental health issues for both trusts as a whole. In April 2020 in Trust A it was the second most common cause of sickness, but by November 2020 it was the most common cause. It was again reported as the most common cause in May and November 2021.

In direct response to national concerns on the impact of the epidemic on health care workers, Trust A instituted several support mechanisms fairly early in the pandemic including individual wellbeing conversations, telephone helplines, opportunities for face-to-face counselling, an online portal with peer-to-peer and personal resilience support and bereavement counselling. However, the qualitative data suggest that staff in both trusts were struggling at the time of the qualitative interviews due to low staffing and the changing nature of their job. What is clear from the qualitative data is that staff continued to strive to provide safe and personalised care when they had the resources to do so, and some went above and beyond even without sufficient resources. However, this is not sustainable and does not provide individualised, person-centred care for the majority of women and their families.

I think, you know, from my point of view, I think our maternity services need to look on maternity. Bosses need to look at the providing of the staffing ratios per patient because I think you know the term, what is it now, making gold out of sows ear, you know comes to mind because you can’t give such a high standard of care with minimal staff, and I think, I can see it now more than before the staff are absolutely exhausted because working with all these PPE is tiring … And I think, you know, especially with young junior midwives that are not getting the support they need, they will burn out or leave the profession. And that’s what bothers me. Health care provider, Trust B.

We need bums on seats because we never get to sit, particularly long, to be able to provide them and spend the extra time, happy face to face appointments, go to women’s houses, see who they are, be available to them rather than this need to get as many in, as many out as we can, because that’s all the time we’ve been given and, have those facilities to be able to them to get the scans when they need them and to be able to see their own midwife and things like that. I’m probably being idealistic, but that’s certainly not the job I wanted when I applied those years ago to be a midwife, I could be there and have a relationship with the women and the families and just make them feel that they were well looked after and that I care. Healthcare provider, Trust A.

## Discussion

This study demonstrates complex and wide-ranging, systems-level changes in demand and processes. It demonstrates limited changes in clinical outcomes, but marked changes related to user experience and staff wellbeing outcomes. It adds to the existing literature by looking more broadly at multiple factors across the maternal health system and documenting the breadth and implications of changes, as well as possible drivers and pathways through which change happened.

### Continuation of services, but adaptation and change

Despite major challenges, mainstream maternity and neonatal services continued to be provided, although some adaptations caused challenges. Lack of strong evidence of poor physical outcomes to mother or baby could reflect that most indicators are unlikely to be responsive to relatively short-term changes as they measure rare events. However, it is also worth noting that the fact that staff consistently “went the extra mile” in extremely difficult circumstances [11] may have ensured that women continued to receive safe care.

Maintaining maternal health was often at considerable cost to staff, who were overstretched even before the pandemic. Qualitative interviews showed examples of women feeling that they had received excellent care, with staff going above and beyond what could be expected of them, and there was evidence that staff reported a strong sense of commitment and purpose. Clearly, however, there were areas where user experience was adversely affected, and services were adapted in ways that both users and staff found challenging. The key three areas for this were the move to remote AN contacts, reduced PN support (particularly in the home) and restrictions on companionship and neonatal visiting.

In contrast to the routine data, the incident reports from Trust A suggest a marked increase in undiagnosed SGA at this time. It is possible that introducing an intervention (such as remote ANC) aimed at promoting safety for service users and staff by reducing the risk of infection can carry other unforeseen risks that should be studied in more detail, and this should be a key area for further research. Similarly, adverse effects on PN care were seen, with concerns raised about the impact on breastfeeding and support more generally. This could be partly responsible for the increased PN admissions for both mothers and babies noted in Trust A’s incident reports (although we cannot provide evidence to support this), and again this could be a useful area for further research. The impact of the pandemic on PN care has been an under-researched area in the UK and requires more analysis. [28, 29].

The importance of maintaining personalised care in maternity services, including during the pandemic, has been emphasised [29]. However, much of the negative feedback relates to a lack of personalisation, including in the user and staff interviews around companionship. There are concerns that restrictions on companionship during ultrasound scans, and in early labour are persisting in some trusts, even though the acute phase of the pandemic appears to be over. Such restrictive policies should be reviewed both now and in anticipation of future crises to ascertain the unintended consequences, and how best to create a balance between reducing infection (or other adverse events, depending on the crisis) and supporting women’s choices [30].

The changes to the way services were delivered were driven by national and trust policy, as well as individual or collective behaviour by health care staff (including management) and service user behaviour change and preference. This complexity may partly explain the difference in responses between the two trusts regarding some aspects of care provision, which is possible due to the level of autonomy given to trusts and practitioners in the UK. This is particularly notable for home births and inductions. One trust responded to national guidelines, while the other did not. These differences are reflected in a national study that found the majority of Trusts in the UK made no major changes to induction practices during the COVID-19 pandemic [31]. National evidence also confirms the temporary cessation of home births in many areas [5].

In many instances, changes made during the initial response to the crisis had not returned to the pre-pandemic situation by the end of our data series (late summer 2021). While trusts may still be facing specific challenges, it is imperative that adaptations such as restricting companionship or telehealth do not become the norm for future practice, just on the basis that they may make the delivery of services easier and/or more efficient for the organisation [32]. Any such changes require rigorous evaluation of negative implications, and a will for rapid de-implementation of processes that are no longer fit for purpose [13].

### Magnification of existing challenges as well as the creation of new ones

One of the particular areas where challenges were identified was staffing. However, it is notable that staffing shortages had existed pre-epidemic, and at the start of the first wave of COVID-19 the situation actually improved, as staff came back from retirement, and the final year student workforce was mobilised. There continued to be no evidence of staffing shortages in the two trusts concerned during the second wave of COVID-19 in Autumn 2020, suggesting that short-term sickness was not impacting significantly on personnel. A marked decline started in May 2021, coinciding with an increase in COVID cases. This decline did not reverse when COVID cases reduced, suggesting that the resilience shown during the first year of the pandemic was wearing out. Both quantitative and qualitative data suggest a more sustained and deep-rooted declinethat is partly driven by the challenges staff have faced throughout the epidemic. Specifically the qualitative evidence points to increasing retirements and disillusionment as a continuation of an ongoing pattern entrenched before the pandemic. This concept of magnification has been a key finding of the ASPIRE COVID-19 project more broadly. It must also be acknowledged that midwifery shortfalls were occurring in the context of other staffing restrictions: the Royal College of Obstetricians and Gynaecologists highlight the widespread redeployment of both junior and senior Obstetricians and Gynaecologists as well as significant levels of absence due to sickness or vulnerability [33].

There were also known and deep-rooted problems with PN care pre-pandemic, that became magnified (and continued to deteriorate) during the pandemic period. Our data suggest that the number of PN visits was declining before the pandemic and this trend continued during the pandemic period. The lack of focus on personalised care found in the case study has also been widely critiqued pre-pandemic. The Friends and Family Test has been the main tool for gathering user feedback within the NHS but has been criticised as being generally inadequate [34]. However, even this poor system was suspended during most of 2020 due to the pandemic, and uptake has not reached anything like pre-pandemic levels now it has been reinstated.

### Critique of the case study methodology for evaluating changes during crisis

The case study methodology enabled us to gather detailed information across time on the dynamic changes which can be compared across trusts. A strength of this study is the integration of quantitative and qualitative data, which allows different perspectives to be triangulated. Quantitative data were limited in some respects, particularly around indicators for personalisation (e.g., companionship). In many cases, the quantitative and qualitative findings complemented each other (e.g., reduction in PN support) but in a few areas, there was some discrepancy: the most notable was remote ANC contact, where quantitative data showed a very small proportion of contacts were not face-to-face meetings, while a number of qualitative comments indicated that face to face contact was perceived by service users to be very limited. This could reflect either a limitation in the quantitative data, or of the qualitative data, that may have highlighted particularly negative experiences or users who had an unusually high number of remote contacts. However, it must be recognised that the data from qualitative interviews may not be representative of users and care providers as a whole as it is based on those who volunteered to take part in the study, who may be a biased sample.

Generally, there were problems with identifying trends in routine data due to missing values, low numbers and general variability for some indicators. These problems were also identified in the other five trusts involved in ASPIRE COVID-19. In some cases, data were not available for a sufficient period before the pandemic to ascertain whether any changes were likely to be attributed to natural variation over time or COVID-related. In particular, there were far fewer data around personalisation than clinical outcomes and safety. Data on continuity of care and the implementation of personalised care packages are routinely collected and reported to the National Maternity Services Dataset but, inconsistency of reporting made this difficult to use.

User and staff experiences are not well represented in the quantitative data, and this is an issue found more widely in UK trusts. Their perspective is critical in understanding the impact of changes either directly or indirectly resulting from the pandemic. In particular, the consistent and regular collection of staff experience is vital in understanding potential deterioration in service delivery, as well as a decline in working conditions. With the current national maternity staff shortages, mechanisms must be set up to consistently collect feedback and act on staff experience, as well as gather their suggestions for service improvement.

Overall, the ASPIRE framework worked well as a method of structuring findings and allowing comparison. We suggest it could be a useful tool in understanding the impacts of shocks and crises on maternal health services more widely than the COVID-19 pandemic and could help structure studies of the impact of adverse events on service delivery, as well as improving routine care throughout the process.

## Conclusion

The case study approach identified widespread direct effects how maternity care was delivered as a result of the pandemic. Deterioration of both AN and PN care, a lack of personalised care and restriction on birth companionship emerged in particular. The quantitative data suggest that problems were present pre-pandemic, leading to a magnification of effect once the crisis took hold. Changes to service uptake and delivery were influenced by national and trust guidelines, as well as staff and user behaviours. There were marked differences in the way the two trusts responded to some situations. Some adaptations introduced early in the pandemic were continued beyond the acute stage of the crisis, with a possibility that they may become the new norm. There were also some unexpected benefits such as easier and expedited procedures for procuring resources, that freed up staff time that was usually engaged in bureaucratic processes.

The impact on user experience could only really be identified from qualitative data as data tracking user experience were unavailable, or unreliable. Additionally, the effects on staff have been considerable. Even after the peak of the pandemic, staff continue to struggle and experience personal sacrifice, mainly due to low staffing and the changing demands of their jobs.

Results were obtained across the entire maternity and neonatal care provision spectrum in two trusts, based on a framework of possible pandemic effects. They suggest that services continued throughout the pandemic, albeit with adaptation and change, yet challenges that pre-dated the pandemic were magnified. This is clear evidence of the need for strong health system components to be in place before new crises and pandemics arise—especially related to staffing, staff and patient wellbeing, and data collection, review and response. Safe and personalised care can still be delivered, even in a crisis, but the stresses on the health infrastructure, especially on staff, should not be underestimated. The ASPIRE COVID-19 framework proved to be a valuable tool in structuring the extensive data gathered and mapping changes in different areas of service provision and may be useful in identifying and tracking changes in the delivery of safe and personalised care in future emergencies or crises, as well as in less critical situations.

## Supplementary Information

**Additional file 1:** **Appendix 1. **Quantitative variables requested for the case study (not all were available in both Trusts). **Appendix 2. **Methodological details for qualitative interviews. **Appendix 3.** Number of reported readmissions of baby and mother based on incident reports (IR1s), quarterly totals for Trust A. **Appendix 4.** Number of reported incidences of undiagnosed SGA, Trust A IR1s (three monthly average). 

## Data Availability

All relevant quantitative and qualitative data will be openly available from the UK Data Service’s online data repository ReShare. Digital object identifiers for each dataset will be provided on paper acceptance.

## Abbreviations

AN: Antenatal
ANC: Antenatal care
CQC: Care quality commission
NHS: National health service
PN: Postnatal
PPE: Personal protective equipment
SGA: Small for gestational age
