# Supplementary material for: Assessing safe and personalised maternity and neonatal care through a pandemic: a case study of outcomes and experiences in two trusts in England using the ASPIRE COVID-19 framework
Source: BMC Health Serv Res. 2023 Jun 22;23:675. doi: 10.1186/s12913-023-09669-0 (PMC10288742; doi:10.1186/s12913-023-09669-0)
Supplement: Supplementary file 1 — Additional file 1: Appendix 1. Quantitative variables requested for the case study (not all were available in both Trusts). Appendix 2. Methodological details for qualitative interviews. Appendix 3. Number of reported readmissions of baby and mother based on incident reports (IR1s), quarterly totals for Trust A. Appendix 4. Number of reported incidences of undiagnosed SGA, Trust A IR1s (three monthly average). [file 12913_2023_9669_MOESM1_ESM.docx]

**Appendix 1**

**Quantitative variables requested for the case study (not all were available in both Trusts)**

| **Domains** | **Examples of indicators requested / collected** |
| --- | --- |
| **Inputs: Demand / need and resources** |  |
| Changes in demand: | Bookings, births, bookings by: deprivation decile, complex social factors, parity and age. |
| Changes in levels of need as direct result of COVID-19 | Regional case numbers, hospital admissions |
| Changes in level of need as indirect result of COVID-19 | % women with mental health concerns at booking, referred for mental health concerns |
| Changes in resources | Staff numbers / midwife ratios, sickness / isolation data |
| **Processes and outputs for safe and personalised care** |  |
| Antenatal care | Gestational age at booking, number of ANC contacts, Personalised care plans, Continuity of care |
| Intrapartum care | Place of birth, type of birth, length of labour, induction rates, pain relief, time to C section, 1-1 care in labour, skin to skin |
| Postpartum care | Breastfeeding, number of postnatal contacts |
| **Outcomes** |  |
| Maternal, fetal, and neonatal mortality, morbidity and wellbeing | Maternal and neonatal mortality, stillbirth, miscarriage, gestational age at birth, PPH, |
| Service user experience | Complaints and IR1s  Any other data collected by trusts (note – none identified) |
| Staff wellbeing and safety | IR1s  Any other data collected by trusts (note – none identified) |

**Appendix 2: Methodological details for qualitative interviews**

**Research questions**

The qualitative interviews used in this study were designed to contribute to a work package designed to answer the following research questions:

·       What organisational changes did NHS Trusts make in response to the COVID-19 pandemic?

·       How and what did NHS Trusts communicate regarding the changes made during the pandemic, to both service users and staff?

·       What are the views and experiences of staff across all levels of the maternity and neonatal services in relation to the organisational response during the pandemic?

·       What are the views and experiences of service users in relation to the organisational response to the pandemic?

·       What are the maternal and neonatal outcomes as reported by organisations during the pandemic?

**Recruitment and sampling**

Participants were identified using a sampling framework:

- Heads of services (up to 10 in each trust)
- Staff (25 in each trust, including midwives, specialist staff, obstetricians, neonatal nurses, Maternity Voice partners etc.).
- Service users (20 in each trust, considering variations in parity, ethnicity, age and COVID-19 status)

Sampling was purposive. For heads of service and other staff, an email, information sheet and consent form were forwarded (electronically) to all staff in the relevant job roles in each participating Trust with a request to respond within two weeks. Service users were recruited through Maternity Voices Partnerships and Trust social media channels as well as respondents from the Babies Born Better survey who expressed an interest in further involvement. There was also targeted recruitment of service users with complex needs through provision of invitations and recruitment packs on maternity wards by research midwives, which were then followed up post-discharge by community midwives.

**Consent**

The information sheet and consent form were reviewed at the start of the interview and then, if the participant was is still willing to proceed, it was signed by the researcher on the participant’s behalf. The consent procedure was audio and video recorded (including participant’s responses and agreement) for verification purposes.

**Semi-structured Interviews:** Interviews were held via Microsoft Teams. Experienced qualitative maternity care researchers and research midwives trained in qualitative research undertook the interviews. Interviewers were allocated to avoid any conflict of interests (i.e. a research midwife would not interview a woman she may have encountered in clinical care, or a colleague in the same Trust). A semi-structured tool was used for the interviews.

For staff, the interviews explored what was normative, easy or difficult to achieve, and what they felt had been beneficial to them, their colleagues, service users, and the organisation, in line with behavioural change theory. This included:

- their experience, involvement and perceptions of who, how, why and what decisions have been made in maternity and neonatal care organisation and provision since February 2020
- how information about service changes have been communicated, monitored and assessed
- what are/have been the likely effects of the service changes, in their view: and
- facilitators and barriers to safe and personal maternity care that they have experienced.

For service users the themes were similar, and covered:

- the participant’s experience, involvement and perceptions of who, how, why and what decisions were made about their care
- how information about service changes were communicated, monitored and assessed
- what were the likely of impacts the service changes, and
- facilitators and barriers experienced to their personal perception that their care was personalised to their values, expectations and beliefs, and safe for them and their baby.

All Interviews were transcribed in vivo by voice-to-text software SONIX and uploaded to MAXQDA for qualitative data management and anonymisation.

**Data Protection and Patient Confidentiality**

This study was compliant with the requirements of the General Data Protection Regulation (GDPR) and Data Protection Act 2018 (DPA) with regards to the collection, storage, processing and disclosure of personal. All data were stored on the University Network (password protected shared drive). Electronic copies of consent forms were saved in a password protected folder. Files that contain personal or identifiable data (e.g. participants log) were encrypted and transferred using file security protocols.

**Appendix 3**

Number of reported readmissions of baby and mother based on incident reports (IR1s), quarterly totals for Trust A

**Appendix 4**

**Number of reported incidences of undiagnosed SGA, Trust A IR1s (three monthly average)**
